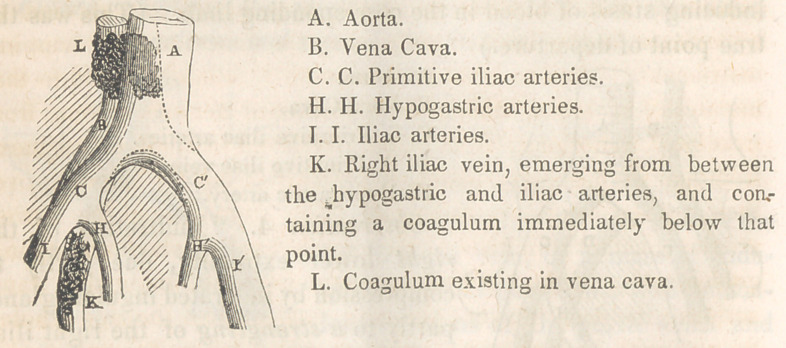# Œdema of the Lower Extremities in Phthisis

**Published:** 1848-05

**Authors:** 


### MEDICAL EXAMINER,

AND

### RECORD OF MEDICAL SCIENCE.

NEW SERIES.—No. XLI.—MAY, 1848.

### ORIGINAL COMMUNICATIONS.

*Œdema of the lower extremities in Phthisis.* Extract of a letter from F. W. Lewis, M. D., to Professor Mütter, dated Paris, December 14th, 1847.

There are many points relative to the anatomy of the human body, which, physiologically considered, present but little to attract attention, yet which from the important part they bear in certain pathological conditions of the economy, become not only highly interesting, but even exercise considerable influence in practical medicine.

The subjoined *cases,*—which all but one were observed and recorded by me, are eminently illustrative of this fact, and serve to demonstrate the importance of more extended researches into the pathology of dropsy than have hitherto been attempted.

That disposition to serous infiltrations, which is so frequently observed during the march of chronic diseases of the great viscera and blood vessels, especially where great mechanical obstruction of the circulation exists, has, not unfrequently, been remarked in these same affections, where no *appreciable* lesion sufficient to account for the dropsical complication was present; and in these cases, to cover an imperfect diagnosis, the perplexed pathologist is apt to invoke a convenient serous diathesis in his patient, or the dropsy is referred to morbid conditions of the circulating fluid. This is especially true in those circumscribed infiltrations of the lower extremities, which occur towards the termination of pulmonary affections, and of various adynamic fevers, where great emaciation has supervened. In many of these cases, it has not escaped attention that the effusion is sometimes confined to a *single limb*, and that where this occurs, that the *left lower extremity* is almost always the seat of the infiltration; a very curious fact, which pathologists have heretofore been contented to pass over in silence, without attempting to assign for it a satisfactory cause, (unless, perhaps, we except Cruveilhier, who in his "Anatomy" hazards an explanation, which the experience of another has recently proved to be nearly the true one.) To M. Piedagnel (Médecin des Hôpitaux) is unquestionably due the merit of having first clearly demonstrated, that the above lesion depends on a mechanical obstruction of the iliac vein, of either side, (usually of the left) by its corresponding artery, or by both, in a manner which the following cases will sufficiently explain.

*Observation* 1. The subject of this observation, a woman aged some 50 years, was admitted about six weeks since into the Hospital, (Notre Dame de la Pitié, service of M. Piedagnel,) at that time suffering under phthisis, during the progress of which she had become frightfully emaciated. She died this morning, (Nov. 13th) when an autopsy was accordingly made.

I had seen this patient sometime previous to her decease, and at the time M. Piedagnel directed our attention to the remarkable circumstance, viz: that the left lower extremity was the seat of a considerable serous infiltration, whereas no analogous condition existed in the right leg, nor indeed, in any other part of the patient's body.

Of this phenomenon M. Piedagnel gave us the following explanation: "When," said he, "during the course of any wasting disease, an individual becomes exceedingly emaciated, the fat which surrounds the aorta and its primitive divisions being absorbed along with that existing elsewhere, the primitive iliac vein, (left), which has previously been separated from the vertebral column by an interposed layer of adipose tissue, is brought into direct contact with the bony structure, at a point immediately opposite the fourth lumbar vertebra, (sometimes the third), against which it is forcibly compressed by the primitive iliac arteries.

This results from peculiar anatomical relations existing between the veins and arteries, (iliac.) Thus whilst the right iliac vein pursues a course nearly parallel with the right iliac artery, and thus altogether escapes compression; on the other hand, the left iliac vein, in passing between the left primitive iliac artery and the spinal column, is pressed against the resisting bodies of the vertebra by the former—an interruption to the circulation in the vein follows—a coagulum forms, and dropsical infiltration of the corresponding lower extremity must inevitably result.

The autopsy revealed the following facts:

As M. Piedagnel had anticipated, the left primitive iliac vein was found forcibly compressed by both the primitive iliac arteries, and immediately below the point of compression existed a consistent semi-organized coagulum, which extended as far down as the beginning of the femoral vein, while evident traces of previous inflammatory action were found, in lymphous adhesions and rough deposits, on the internal parietes of the vein. The iliac and femoral veins of the right side on being opened were found to contain little or no blood.

The disposition of the iliac veins and arteries, relatively to each other and the vertebral column, was as represented in the following sketch.

In this case the division of the aorta into its primitive iliacs took place immediately opposite the third lumbar vertebra, and the position of the vena cava was considerably farther to the right than is usually the case.

A. Aorta.

B. Vena cava.

C. C. Right and left primitive iliac arteries.

D. D. Right and left primitive iliac veins.

E. E. Hypogastric arteries.

F. Point of compression.

2. and 3. Second and third lumbar vertebra.

*Observation* 2. "Infiltration of the left lower extremity due to compression of the left primitive iliac vein, by its corresponding artery."

A woman having died of phthisis in the Hospital, (La Pitié,)

service of M. Piedagnel, an autopsy was made on the morning of the 31st October, 1847. I had not seen this case previous to her decease, but M. Piedagnel observed to us, before opening the body, that a dropsical infiltration of the left leg, precisely similar to that observed in the preceding case, had existed for a considerable time before the patient's death; calling our attention at the same time to the swollen condition of that limb as contrasted with the frightful emaciation of the right lower extremity, and the posture of the body. The abdomen being opened, the aorta was found to divide into the primitive iliac arteries, immediately over the point where the two iliac veins unite to form the vena cava ascendens, and directly opposite the fourth lumbar vertebra. As in the preceding case, the left iliac artery, in crossing, strongly compressed the primitive iliac vein of the same side, against the vertebral column, but owing to a more normal arrangement of the vessels, the right iliac artery (as in that instance) did not participate in causing the pressure.

The vein on being opened throughout its length was found to be filled completely with very dark coloured coagula of blood—coagula more or less consistent—and evidently of long formation. These were in places adherent to the parietes of the vein which gave evidence of having been inflamed. Similar adherences with an analogous colouration of the serous lining, were discovered extending along the femoral almost to its termination. No similar phenomena were observable on the right side, its veins being comparatively free from blood.

*Figure, showing the relations existing between the iliac arteries and veins in the foregoing case.*

A. Aorta.

B. Vena Cava.

C. C. Right and left iliac arteries.

D. D Right and left iliac veins.

E. Coagulum existing on left side.

*Observation* 3. " Compression of the left femoral vein, by a mass of indurated inguinal ganglions, with anomalous relations of the pelvic veins ,and arteries."

A man aged 38, was admitted into the Hospital La Pitié, wards of M. Piedagnel, suffering under a slow chronic dysentary,

(contracted on the island of Martinique,) during the course of which disease he became frightfully emaciated; to such an extent indeed, that his coxal bones nearly protruded through the skin ; while his body much resembled a skeleton, tightly covered with parchment, with the exception, however, of the left lower extremity, which presented a remarkable difference in this respect, it being the seat of a considerable dropsical infiltration, unusually plump and rounded. M. Piedagnel judged that in this case, as in the two previously observed, a compression of the left iliac vein, by its corresponding artery, would be found to exist. Such, however, did not prove to be the fact, the relation of the arteries to the veins being such that the left primitive iliac vein was abruptly crossed opposite the last lumbar vertebra by the hypogastric artery ; but in no part of its course was this vein traversed by the primitive iliac artery of the same side, which vessel pursued a course absolutely parallel with it. The artery in this case was considerably in advance of the vein. But in crossing the left primitive iliac vein the hypogastric artery finding no point d'appui against the vertebral column, little or no compression was exercised on the former, at least no compression sufficiently powerful to interfere with a due return of venous blood to the vena cava; consequently, no fibrinous clot existed in the vein. In the femoral vein of the same side, however, at a point immediately above where that vessel emerges from beneath Poupart's ligament was found the cause of the obstruction, viz.: numerous indurated inguinal ganglions directly compressing the vein, and thus inducing stasis of blood in the corresponding limb. (This was the true point of departure.)

A. Aorta.

B. Vena Cava.

C. C. Primitive iliac arteries.

D. D. Primitive iliac veins.

H. Hypogastric artery.

*Observation* 4. "Infiltration of the *right* lower extremity, due partly to compression by indurated inguinal gland, partly to *a strangling* of the right iliac vein by the right iliac and hypogastric arteries."

The patient, a man aged 40, died of a long protracted phthisical affection, for which he had been admitted into the Hospital La Pitié some weeks since. Unlike the previous cases, for some time before his death his *right* leg was observed to be the seat of dropsical effusion, the left extremity being in no ways affected similarly. The autopsy being made, the indurated inguinal ganglions were found surrounding the origin of the right crural vein, below which point fibrinous coagula of recent formation were discovered, the internal surface of the vein being rough and unpolished, and in some places deeply stained. Another coagulum of considerable density, and apparently long formed, existed in the origin of the vena cava, at a distance of about an inch above the division of the aorta, resting in a kind of *aneurismal* sac, constituted by a dilatation of the vein. Above this point, up to which the left primitive iliac freely carried its blood, no obstruction appeared to exist, nor could the existence of this firm semi-organized clot be readily accounted for. A third coagulum, also of ancient formation, was found to exist in the right primitive iliac vein, at a point intermediate between its origin and its termination in the vena cava. In this instance, pursuing a serpentine course, the vein passing at first from right to left, and from behind forward, wound its way between the right iliac and hypogastric arteries, in a manner which the rough sketch annexed will serve to illustrate. From these unnatural relations of the right iliac vein, more or less strangling of that vessel and consequent obstruction to the circulation undoubtedly ensued.

## Figures and Tables

**Figure f1:**
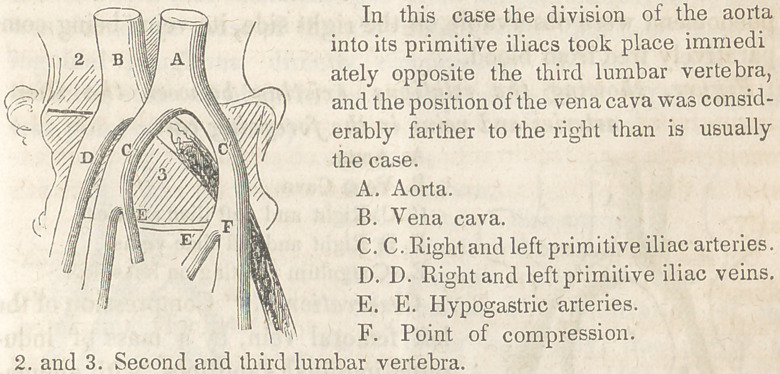


**Figure f2:**
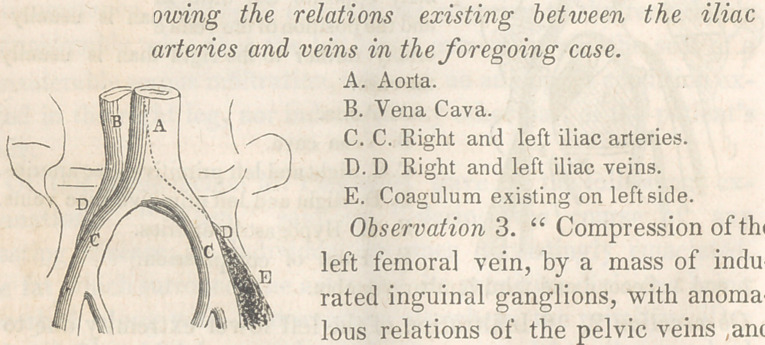


**Figure f3:**
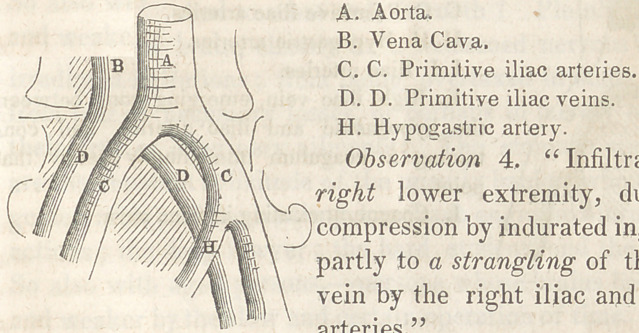


**Figure f4:**